# Sphingosine 1‐phosphate and its carrier apolipoprotein M in human sepsis and in *Escherichia coli* sepsis in baboons

**DOI:** 10.1111/jcmm.12831

**Published:** 2016-03-17

**Authors:** Cecilia Frej, Adam Linder, Kaisa E. Happonen, Fletcher B. Taylor, Florea Lupu, Björn Dahlbäck

**Affiliations:** ^1^ Department of Translational MedicineDivision of Clinical ChemistrySkåne University HospitalLund UniversityMalmöSweden; ^2^ Department of Clinical SciencesDivision of Infection MedicineLund UniversitySkåne University HospitalLundSweden; ^3^ Cardiovascular Biology Research ProgramOklahoma Medical Research FoundationOklahoma CityOKUSA

**Keywords:** HDL, SIRS, apolipoproteins, lysophospholipids, lipoproteins

## Abstract

Sphingosine 1‐phosphate (S1P) is an important regulator of vascular integrity and immune cell migration, carried in plasma by high‐density lipoprotein (HDL)‐associated apolipoprotein M (apoM) and by albumin. In sepsis, the protein and lipid composition of HDL changes dramatically. The aim of this study was to evaluate changes in S1P and its carrier protein apoM during sepsis. For this purpose, plasma samples from both human sepsis patients and from an experimental *Escherichia coli* sepsis model in baboons were used. In the human sepsis cohort, previously studied for apoM, plasma demonstrated disease‐severity correlated decreased S1P levels, the profile mimicking that of plasma apoM. In the baboons, a similar disease‐severity dependent decrease in plasma levels of S1P and apoM was observed. In the lethal *E. coli* baboon sepsis, S1P decreased already within 6–8 hrs, whereas the apoM decrease was seen later at 12–24 hrs. Gel filtration chromatography of plasma from severe human or baboon sepsis on Superose 6 demonstrated an almost complete loss of S1P and apoM in the HDL fractions. S1P plasma concentrations correlated with the platelet count but not with erythrocytes or white blood cells. The liver mRNA levels of apoM and apoA1 decreased strongly upon sepsis induction and after 12 hr both were almost completely lost. In conclusion, during septic challenge, the plasma levels of S1P drop to very low levels. Moreover, the liver synthesis of apoM decreases severely and the plasma levels of apoM are reduced. Possibly, the decrease in S1P contributes to the decreased endothelial barrier function observed in sepsis.

## Introduction

Sepsis is a leading cause of death worldwide, the yearly US incidence being around 300 cases per 100,000 individuals 1. In sepsis, microorganisms enter the circulation leading to the development of septic shock, disseminated intravascular coagulation and organ failure. The strong acute phase response in sepsis is associated with major changes in the lipid and protein content of the plasma lipoproteins. High‐density lipoprotein (HDL) is more affected than low‐density lipoprotein (LDL) with decreased cholesterol‐ester content and changed protein composition. The plasma concentration of apolipoprotein A1 (apoA1), the major HDL protein, decreases during sepsis and correlates inversely with survival 2, 3. HDL has multiple anti‐inflammatory properties 4, 5 and is part of the innate immune defence. The HDL‐associated lipopolysaccharide (LPS)‐binding protein binds and neutralizes LPS and inhibits LPS‐induced release of tumour necrosis factor (TNF)‐α *in vivo* 6, 7, 8. HDL also exerts endothelial protective effects and stimulates the endothelial barrier function, properties which have been associated with the bioactive lipid sphingosine 1‐phosphate (S1P) 9, 10. In plasma, 60% of S1P is normally bound to the apolipoprotein M (apoM) and the remaining 40% to albumin 11. ApoM is unusual among the apolipoproteins in being bound to the lipoproteins (mainly HDL) *via* its retained signal peptide 12, 13, 14. It is structurally a member of the lipocalin family, having a hydrophobic pocket for specific S1P binding 15. Hepatic overexpression of apoM in mice leads to increased levels of plasma S1P, indicating that apoM is involved in S1P‐homoeostasis 16, 17.

S1P is a lysophospholipid that activates five different G‐coupled receptors, S1P_1‐5_ 18. It is mainly derived from erythrocytes, endothelial cells and platelets. S1P is produced *de novo* from hydrolysis of sphingomyelin, which is converted to ceramide and then to sphingosine *via* sphingomyelinase and ceramidase respectively. Two kinases, sphingosine kinase 1 and 2 (Sphk1 and Sphk2) phosphorylate sphingosine to S1P 19. S1P can be degraded irreversibly by S1P‐lyase (S1PL) or de‐phosphorylated to sphingosine by the specific S1P‐phosphatases 1 and 2 (Sgpp1 and Sgpp2) or by broad targeted lipid phosphohydrolases 20, 21.

S1P is involved in the regulation of cytokine secretion, maintenance of endothelial barrier function, activation of mast cells and migration of immune cells 22, 23, 24, 25, 26, 27. In sepsis, the endothelial barrier function is impaired and the vascular wall becomes leaky leading to decreased blood pressure, contributing to the development of septic shock. S1P increases the trans‐monolayer electric resistance across both human and bovine endothelial cells, mainly *via* S1P_1_ activation 9, 24. The barrier function is enhanced by an induction of cadherin‐containing adherent junctions between endothelial cells following S1P_1_‐stimulation by S1P 14. In patients with dengue fever, a disease associated with endothelial hyperpermeability, S1P levels were decreased in patients with plasma leakage compared to patients with no plasma leakage 28. In addition, S1P‐deficient mice have increased vascular leakage and mortality after anaphylaxis compared to control mice 29 and rats have reduced loss of plasma volume during sepsis after administration of the S1P‐analogue FTY720 30, indicating a role for S1P‐regulated events in the pathology of plasma leakage.

ApoM decreases in both mice and humans during acute inflammation and sepsis 31, 32 and very recently, Winkler *et al*. 33 showed that S1P decreases in human sepsis. By analysing a large patient cohort, we now confirm that the S1P plasma concentration decreases during sepsis, the level of decrease depending on severity of the patients' disease. Moreover, to study in more detail the changes in S1P and apoM during disease progression in sepsis, we have used archived samples from a well‐characterized non‐human primate model of sepsis and analysed factors contributing to alterations in plasma levels of S1P. We observe that the decrease in S1P reflects the severity of the septic disease and that the S1P decrease occurs very early during the septic challenge preceding the drop in apoM concentration.

## Materials and methods

### Patients and controls

The local ethical committee at Lund University granted permission for the human sample collection and analysis (Dnr. 790/2005). The human study included 202 patients enrolled at the Emergency Department at the Clinic for Infectious Diseases, University Hospital, Lund, Sweden 34. Informed consent was obtained from all participants. The initial inclusion criteria were suspected infection, fever (≥38°C) and age above 18 years and the patients were then enrolled based on the following systemic inflammatory response syndrome (SIRS)‐criteria: body temperature ≥38°C, white blood cells count >12 × 10^9^ cells/l or <4 × 10^9^ cells/l, pulse rate >90 beats/min., respiratory rate >20 breaths/min. or hypotension with systolic blood pressure <90 mmHg or a decrease in >40 mmHg from baseline. Within 12 hrs of admission, citrated blood was collected, centrifuged at 2000 × *g* for 10 min. and the plasma frozen at −80°C. White Blood Cell count and platelet count were standard analyses performed at the Clinical Chemistry laboratory at Lund University Hospital. Citrated plasma from 23 healthy volunteers from the hospital staff were collected and processed in the same way as the patient samples. Two independent physicians, unaware of the S1P and apoM results, classified the patients into the following five different groups based on SIRS‐criteria, the presence or absence of organ failure, and final diagnosis: septic shock *n* = 20 (severe sepsis including resistant hypotension), severe sepsis *n* = 44 (criteria for severe sepsis were infectious disease, at least two SIRS criteria and/or development of organ failure or hypotension within 24 hrs after blood sampling), sepsis *n* = 83 (infectious disease with at least two SIRS criteria and absence of organ failure), infection without SIRS *n* = 37 and SIRS without infection *n* = 18 (non‐infectious disease with at least two SIRS‐criteria). All patients with severe sepsis (with or without septic shock) were hospitalized, as were 75% of the patients in the sepsis group, 33% of the patients with infection without SIRS, and 83% of the patients with SIRS without infection. The diagnoses in the SIRS without infection group were pulmonary embolisms, cardiogenic shock and haemorrhagic ulcers and 61% had organ dysfunction.

### Sepsis model in baboons

We used archived plasma and tissue samples from experiments approved by the Institutional Animal Care and Use Committees of both Oklahoma Medical Research Foundation and the University of Oklahoma Health Science Centre (OUHSC). The baboons were between 2–3 years old and weighed 7–10 kg. They were fasted over night and given water *ad libitum*. They were sedated with ketamine hydrochloride (20 mg/kg, intramuscularly) and anaesthetized with sodium pentobarbital, 25 mg/kg every 30 min. or as deemed necessary by monitoring the eyelid reflex. The animals were intubated orally and were breathing freely. For blood sampling and infusion of *E. coli,* the femoral vein and the saphenous vein were cannulated aseptically. The dose of *E. coli* was 10^9^ colony‐forming units (cfu)/kg to induce LD_50_ (*n* = 14) and 1–2 × 10^10^ cfu/kg to induce LD_100_ sepsis (*n* = 8). Infusion of *E. coli* started at time‐point 0 and was then given continuously for 2 hrs. In the LD_50_ group, blood samples were taken before challenge and up to 7 days post‐infusion and in the LD_100_ group before challenge and up to 24 hrs post‐infusion. Complete blood count was done using a VetScan HM5 hematology analyzer (Abaxis, Union City, CA, USA). All animals were sacrificed after completion of the experiments. To study mRNA levels, tissues from liver and kidney were analysed from 23 and 15 baboons, respectively. Of the analysed liver biopsies, 11 belonged to the LD_50_ group, 7 to the LD_100_ group and 5 to the control group, which included animals sacrificed for seizures, a non‐inflammation‐related condition. The kidney biopsies originated from seven baboons in the LD_50_ group, five in the LD_100_ group and three in the control group. Baboons in the control group received saline infusion only and were Sacrificed directly after infusion. Liver and kidney tissues from septic baboons were collected from 2 to 34 hrs post‐challenge.

#### S1P quantification

S1P was quantified as previously described 11. In brief, S1P (d‐*erythro*‐sphingosine‐1‐phosphate) and internal standard (IS) (d‐*erythro*‐sphingosine‐d7‐1‐phosphate) were purchased from Avanti lipids (Alabaster, AL, USA). Plasma was diluted 1:6.5 in TBS (50 mM Tris‐HCl, 0.15 M NaCl, pH 7.5) and precipitated in methanol containing 20 nM IS. Samples were centrifuged at 17,000 × *g* for 2 min. and 5 μl of the supernatants were injected for analysis by liquid chromatography that was coupled to a triple quadropol mass spectrometer (LC‐MS/MS) (API 4000 from Sciex, Framingham, MA, USA). Analytes were separated on a reversed phase C18‐column (XSelect CSH XP C18 130 Å, 2.5 μm, 2.1 mm × 50 mm from Waters, MA, USA) and ionized by Electro Spray Ionization operating in positive ionization mode. Scanning mode was multiple reaction monitoring. The following *m/z* transitions were chosen for quantitative and qualitative analysis, respectively, for S1P 380/264, 380/82 and for IS 387/271, 387/82. Obtained results were integrated and calculated using the Analyst^®^ software (AB Sciex).

### ApoM/ApoA1‐ELISA

ELISAs developed for human apoM and apoA1 were found to function well for the measurements of the baboon counterparts in plasma samples and were performed as previously described 31, 35. Standard curves were prepared from pooled plasma samples taken from the baboons at time‐point zero.

### ApoE quantification

Plasma (1 μl) was analysed by SDS‐PAGE followed by Western blotting (Biorad, Hercules, CA, USA). ApoE was visualized by probing the blots with a rabbit polyclonal anti‐human apoE antibody (nr A00077) followed by a HRP‐conjugated goat polyclonal antirabbit antibody (nr P0448, both from DAKO, Glostrup, Denmark). The blots were developed using ECL in a ChemiDoc MP Imager (Biorad). ApoE levels were normalized against albumin by probing with rabbit antibovine albumin (nr A11133 from Invitrogen, Eugene, OR, USA).

### Analysis of LDL, HDL and albumin

Plasma levels of total cholesterol, LDL and HDL were measured using the EnzyChrom HDL and LDL/VLDL Assay Kit (EHDL‐100; Bioassay Systems, Hayward, CA, USA) and albumin by QuantiChrom BCG Albumin Assay Kit (Bioassay Systems).

### Total RNA extraction from liver and kidney tissues

Liver or kidney tissues weighing 40–60 mg were ground in liquid nitrogen using a mortar and pestle. The tissue was re‐suspended in 1 ml of TRIzol (Invitrogen, Carlsbad, CA, USA) and extraction of RNA was performed according to the manufacturer's instructions. The RNA was further purified by the Qiagen mini RNEasy‐kit (Qiagen, Hilden, Germany).

### mRNA Quantification

To RNA, a mastermix made of 2ROX, Taq‐polymerase (Superscript^™^ III RT Platinum^®^ Taq‐mix 1325133; Invitrogen), probes for apoM (Hs01597780_g1), apoA1 (Hs0098500_g1), apoE (Rh0279929_m1), albumin (Rh02828765_m1), Sgpp1 (Mf04372495_m1), Sgpp2 (Rh00544786_m1), S1PL (Rh00393705_m1), Sphk1 (Hs00184211_m1), Sphk2 (Rh02876562_m1), serum amyloid A (SAA) (Hs00293702_m1) and the housekeeping gene glyceraldehyde‐3‐phosphate dehydrogenase (GAPDH, Rh02621745_g1), (Applied Biosystem, Foster City, CA, USA) were added. qRT‐PCR reactions were performed on an ABI Prism 7900 HT Sequence Detection System (Applied Biosystems) or CFX384 C1000 Thermal cycler (Bio Rad, USA). Changes in mRNA levels were calculated according to the 2^−ΔΔC^_T_ method 36.

### Gel filtration

Plasma was pooled from 10 healthy volunteers and from 10 human patients with severe sepsis (500 μl total volume) and from four baboons at time‐point zero and 48 hrs (LD_50_) or 24 hrs (LD_100_) (total volume 200 μl). The plasma pools were separated on Superose 6 10/300 GL column connected to an ÄKTA AVANT system (GE Healthcare, Uppsala, Sweden). Gel filtration was performed in TBS with a flow rate 0.4 ml/min. and 30 fractions containing 300 μl each were collected.

### Statistic analysis

The statistical significance of differences between the groups in the human sepsis cohort was determined using a Kruskal–Wallis test with Dunn's multiple comparison test. In the baboon cohort, statistical significance of the decrease in analytes between different time‐points was determined by Durbin–Skillings–Mack test. This paired non‐parametric anova was chosen because of the possibility to handle missing data points in the data set that emerged when some baboons had to be sacrificed at early time‐points. Statistical analysis between different time‐points in the mRNA analysis was made by Kruskal–Wallis test with Dunn's multiple comparison test. For evaluating correlations, the Spearman's rank correlation coefficient was calculated. *P* values <0.05 were considered statistically significant. Statistics were calculated in GraphPad Prism 4.0 (GraphPad Software, La Jolla, CA, USA) or XLSTAT 2015.4.01.20578 (Addinsoft, Paris, France).

## Results

### S1P decreases in human sepsis

**Figure 1:**
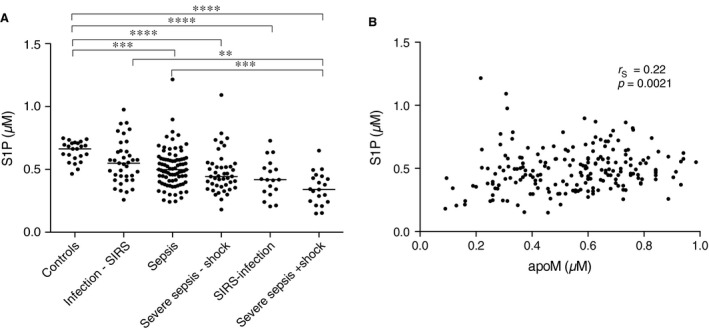
S1P decreases in human sepsis. Human adult patients admitted to the emergency department with suspicion of infection were enrolled in the study based on the systemic inflammatory response syndrome (SIRS)‐criteria. Plasma was collected from patients with severe sepsis with shock (*n* = 20), severe sepsis without shock (*n* = 44), sepsis (*n* = 83), infections without SIRS (*n* = 37), SIRS without infection (*n* = 18) and healthy controls (*n* = 23). Plasma S1P was quantified by LC‐MS/MS (A) and correlated with plasma apoM (B). Statistical analysis was performed with a Kruskal–Wallis test *P < 0.05, **P < 0.01, ***P < 0.001, ****P < 0.0001, *r*_s_ = Spearman's correlation coefficient.

The endothelial protective sphingolipid S1P stimulates the assembly of cadherin junctions between endothelial cells. Since the endothelium becomes leaky in sepsis, we suggested that S1P decreases in sepsis, thus contributing to the endothelial pathology. We therefore quantified plasma S1P in a cohort of 202 patients previously investigated for apoM 31. S1P was decreased in most groups as compared to controls; −46% in severe sepsis with shock (*P* < 0.0001), −34% in SIRS without infection (*P* < 0.0001), −27% in severe sepsis without shock (*P* < 0.0001), −21% in sepsis (*P* < 0.001) and −14% in infections without SIRS (non‐significant) (Fig. 1A). The S1P concentration in patients with severe septic shock was significantly lower as compared to patients with sepsis (*P* < 0.01) and infection without SIRS (*P* < 0.001) (Fig. 1A). These results followed the pattern of plasma apoM, previously analysed in this cohort 31. There was a weak but significant correlation between plasma levels of apoM and S1P in the sepsis patients (*r*_S_ = 0.22, *P* = 0.0021) (Fig. 1B).

### S1P and apoM decrease in non‐human primate sepsis

**Figure 2:**
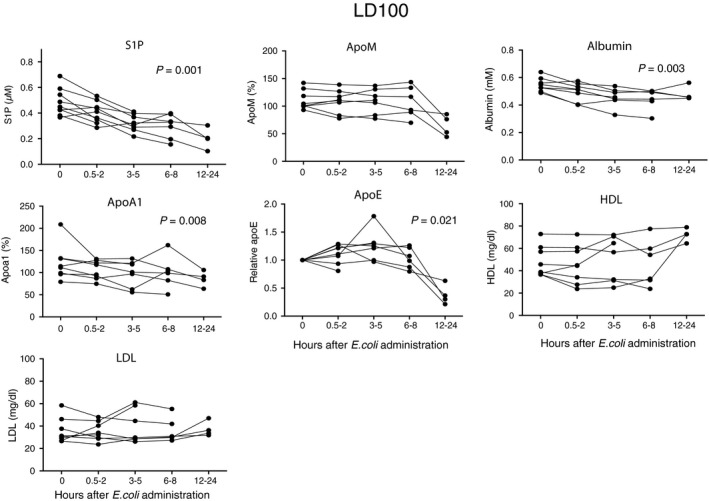
S1P and apoM in lethal sepsis. Baboons (*n* = 8) were challenged with 1–2 × 10^10^ cfu/kg of *E. coli* (LD_100_). Blood samples were collected at intervals from time‐point zero to 24 hrs post‐infusion except for from the baboons that had to be sacrificed earlier. S1P was measured by LC‐MS/MS, apoM and apoA1 by ELISA, albumin, HDL‐cholesterol and LDL‐cholesterol by commercial kits, and apoE by Western blotting (values being given relative to time‐point zero). Data are presented as individual values for each baboon. Statistical analysis of differences between time‐points was performed with a Durbin–Skillings–Mack test.

To study S1P and apoM in further detail, we used a well‐characterized baboon model of sepsis where the disease progression is similar to that of human sepsis 37. By administering two doses of *E. coli*, different severities of sepsis were established, the LD_50_ group where 50% of the baboons survived and the LD_100_ group where all baboons got terminally ill and were sacrificed within 24–36 hrs post‐challenge 38. In baboons administered a high dose of *E. coli* (LD_100_ group), plasma S1P decreased by 59% (Fig. 2) and apoM by 42% (Fig. 2) within 24 hrs. Albumin, the second carrier of S1P, decreased by 12% (Fig. 2). The levels of LDL and HDL were not significantly altered (Fig. 2), although a 29% reduction in apoA1 was seen at 12–24 hrs (Fig. 2). Since there was no effect on the HDL cholesterol despite lowering of both apoA1 and apoM, we analysed apoE, which is reported to be increased in sepsis 39. However, in the LD_100_ group, apoE was strongly decreased at 12–24 hrs (Fig. 2). Serum Amyloid A has been suggested to be increased in HDL during severe inflammation 40, 41 and we hypothesize that SAA could possible replace the other apoliproproteins in the HDL‐particle during an acute phase response in our model. However, we could not detect any SAA in baboon plasma (using anti‐human antibodies in western blotting) and in the liver there was a slight decrease in transcription of SAA (Fig. S1).

**Figure 3:**
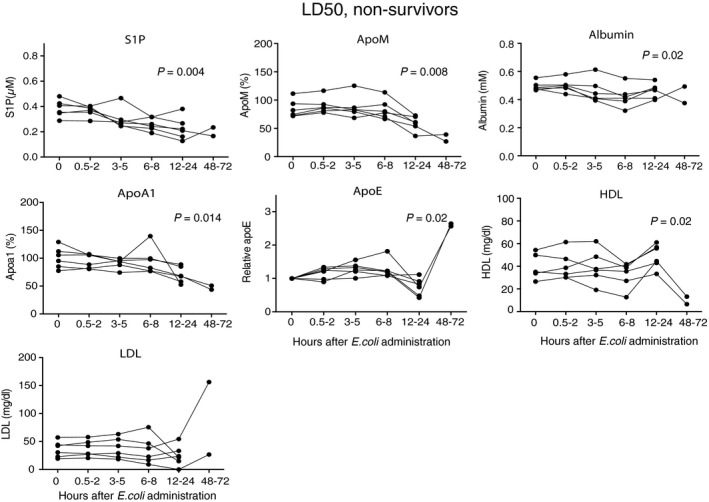
S1P and apoM in non‐survivors in the LD_50_ sepsis‐group. Baboons (*n* = 6) were challenged with 10^9^ cfu/kg of *E. coli* (LD_50_). Blood samples were collected at intervals from zero to 72 hrs post‐infusion except for from the baboons that had to be sacrificed earlier. S1P was measured by LC‐MS/MS, apoM and apoA1 by ELISA, albumin, HDL and LDL were measured by commercial kits and apoE by Western blotting (values being given relative to time‐point zero). Data are presented as individual values for each baboon. Statistical analysis of differences between time‐points was performed with a Durbin–Skillings–Mack test.

**Figure 4:**
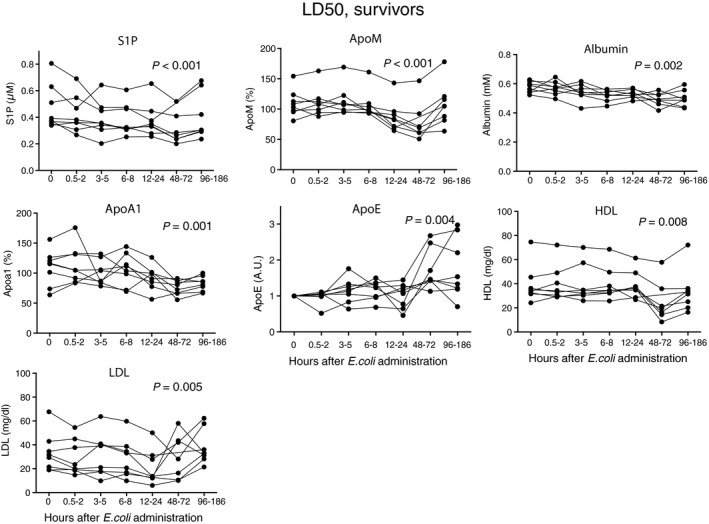
S1P and apoM in survivors of the LD_50_ sepsis‐group. Baboons (*n* = 8) were challenged with 10^9^ cfu/kg of *E. coli* (LD_50_). Blood samples were collected at intervals from zero to 7 days. S1P was measured by LC‐MS/MS, apoM and apoA1 by ELISA, albumin, HDL and LDL were measured by commercial kits and apoE by Western blotting (values being given relative to time‐point zero). Data are presented as individual values for each baboon. Statistical analysis of differences between time‐points was performed with a Durbin–Skillings–Mack test.

In the LD_50_ group, the decrease in S1P and apoM reflected disease severity. S1P decreased significantly in the non‐survivors by up to 56%, whereas a 35% decrease was observed in the survivors (Figs 3 and 4). ApoM correspondingly decreased significantly in both non‐survivors (by 61%) and survivors (by 28%), reaching the lowest levels after 48–72 hrs (Figs 3 and 4). A weaker decrease was observed for albumin, 12% in the non‐survivors and 14% in the survivors after 48–72 hrs (Figs 3 and 4). ApoA1 decreased by 53% after 48–72 hrs in the non‐survivors and by 30% in the survivors (Figs 3 and 4). Similarly to the LD_100_ sepsis group, apoE was decreased in the non‐survivors at 12–24 hrs (Fig. 3). However, in contrast to apoM and apoA1, the initial decrease in apoE was followed by a substantial increase at later time‐points in many but not all animals (Figs 3 and 4). HDL was unchanged at 24 hrs but after 48–72 hrs levels decreased by 74% in the non‐survivors and by 37% in the survivors (Figs 3 and 4). High‐density lipoprotein has been reported to decrease also in human sepsis 42. In contrast, VLDL/LDL decreased at 24 hrs and thereafter increased in both the non‐survivors and survivors (Figs 3 and 4), following the pattern of apoE.

### Decreased transcription of apolipoproteins and albumin in sepsis

**Figure 5:**
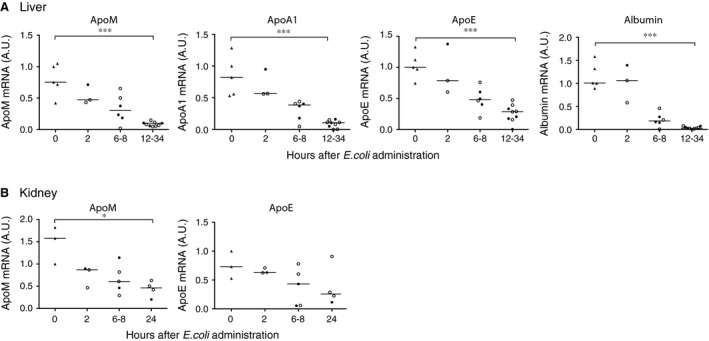
Liver and kidney mRNA levels of apolipoproteins are decreased in sepsis. mRNA from liver (A, *n* = 23) and kidney (B, *n* = 15) tissue was extracted from baboons challenged with 10^9^ cfu/kg of *E. coli* (LD_50_, open circles) or 1–2 × 10^10^ cfu/kg (LD_100_, closed circles) dose of *E. coli* or saline solution alone (triangles). Baboons receiving the saline infusion were sacrificed directly after the infusion (*t* = 0 h). Tissue samples from the liver in septic baboons were taken after 2 (*n* = 3), 6 (*n* = 1), 8 (*n* = 5), 12 (*n* = 1), 24 (*n* = 6), 27 (*n* = 1) and 34 (*n* = 1) hours and from the kidney after 2 (*n* = 3), 6 (*n* = 1), 8 (*n* = 4) and 24 (*n* = 4) hours. mRNA levels were measured by qRT‐PCR, normalized against GAPDH and are given relative to one reference baboon. Statistical analysis was performed with Kruskal–Wallis test, *P < 0.05, ***P < 0.001.

Apolipoproteins are mainly synthesized in the liver although both apoM and apoE mRNA are present in the kidney 12, 43. To study whether the decrease in plasma concentrations of apolipoproteins was associated with reduced production, qRT‐PCR analyses of liver and kidney biopsies were performed. In both the LD_50_ and LD_100_‐groups, the mRNA levels of apoM and apoA1 were decreased substantially in the liver already after 6–8 hrs, reaching the lowest values at 12–34 hrs (Fig. 5A). Despite an increase in plasma levels of apoE in the LD_50_ sepsis‐group after 24 hrs (Figs 3 and 4), the transcription of apoE was strongly decreased in the liver at 12–34 hrs, which was the latest time‐point where tissue samples were available (Fig. 5A). There was a similar but less pronounced decrease in mRNA levels of apoM and apoE in the kidneys (Fig. 5B). The transcription of albumin was strongly decreased in the liver (Fig. 5A). The long half‐life of albumin (e.g. up to 19 days 44) explained that the plasma albumin levels were fairly stable (Figs 2, 3, 4).

We also evaluated the liver mRNA‐levels for enzymes involved in the S1P metabolism, for example, Sphk1, Sphk2, Sgpp1, Sgpp2 and S1PL, even though the liver is not a major organ contributing to circulating plasma S1P and the changes observed are therefore of uncertain significance for the S1P levels. qRT‐PCR analysis of liver mRNA showed that transcription of the S1P‐generating Sphk2 mRNA was decreased already after 2 hrs compared to controls (Fig. S1), whereas Sphk1 was transiently up‐regulated, reaching a peak after 6–8 hrs (Fig. S1). Sgpp1 was unaffected at early time‐points and mildly decreased at 12–34 hrs, whereas Sgpp2 was transiently increased after 6–8 hrs and stayed above basal levels at 12–34 hrs (Fig. S1). mRNA levels of S1PL were down‐regulated after 2 hrs and stayed low after 12–34 hrs (Fig. S1).

### S1P levels correlate with bacterial count in plasma

The number of circulating live bacteria measured 2 hrs after start of the *E. coli* infusion correlated significantly to the decrease in S1P at 24 hrs (*r* = 0.55, *P* = 0.02) (Fig. S2) indicating that the decrease in S1P correlated with the severity of the bacteraemia. The correlation between the bacterial count and the decrease in apoM and apoA1 did not reach statistical significance (Fig. S2). No correlation was found between albumin changes and bacterial count (Fig. S2).

### S1P correlates with platelet counts but not with erythrocytes and white blood cells

**Figure 6:**
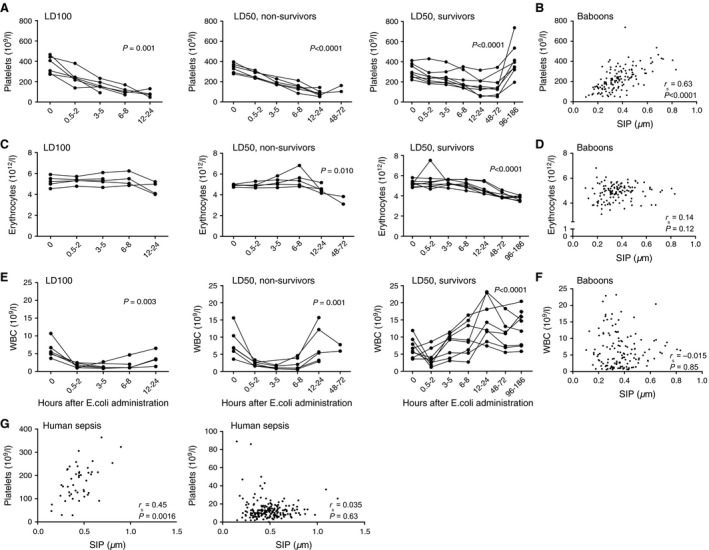
S1P correlates with platelets but not with erythrocytes or white blood cells. Baboons were challenged with 10^9^ cfu/kg of *E. coli* (LD_50_) or 1–2 × 10^10^ cfu/kg (LD_100_) dose of *E. coli* (*n* = 22) and blood was drawn over time. Human adult patients admitted to the emergency department with suspicion of infection were enrolled in the study based on the SIRS‐criteria. Correlation of S1P with blood cell counts was calculated from all data points in the baboon cohort and from available data points in the human sepsis cohort. (A) Platelet distribution in baboons. (B) Correlation between platelets and S1P in baboons. (C) Erythrocyte distribution in baboons (D) Correlation between erythrocytes and S1P in baboons. (E) WBC distribution in baboons. (F) Correlation between WBC and S1P in baboons. (G) Correlation between platelets (right figure), WBC (left figure) and S1P in human sepsis patients. Statistical analysis of differences between time points was performed with Durbin–Skillings–Mack test. *r*_s_ = Spearman's correlation coefficient.

In the baboon experiment, plasma apoM decreased later than S1P, which suggests that it is not the loss of apoM that causes the decrease in S1P. S1P is produced mainly by erythrocytes and platelets in human blood and during sepsis blood cells are highly affected 45, 46. We therefore analysed the relation between blood cells and S1P in both humans and baboons. Platelets decreased rapidly upon sepsis induction in the baboons (as described before 47) (Fig. 6A), whereas erythrocytes decreased at later time‐points (after 6–8 hrs) but were only mildly affected (Fig. 6C). The white blood cells decreased directly upon sepsis induction in all sepsis groups but increased at later time‐points (Fig. 6E). S1P correlated significantly with platelet counts in both baboons (Fig. 6B) and in humans (6G). However, S1P did not correlate with erythrocyte levels in baboons (Fig. 6D) or with white blood cells in either baboons (Fig. 6F) or humans (Fig. 6G).

### Decrease in apoM‐associated S1P during sepsis revealed by gelfiltration chromatography

**Figure 7:**
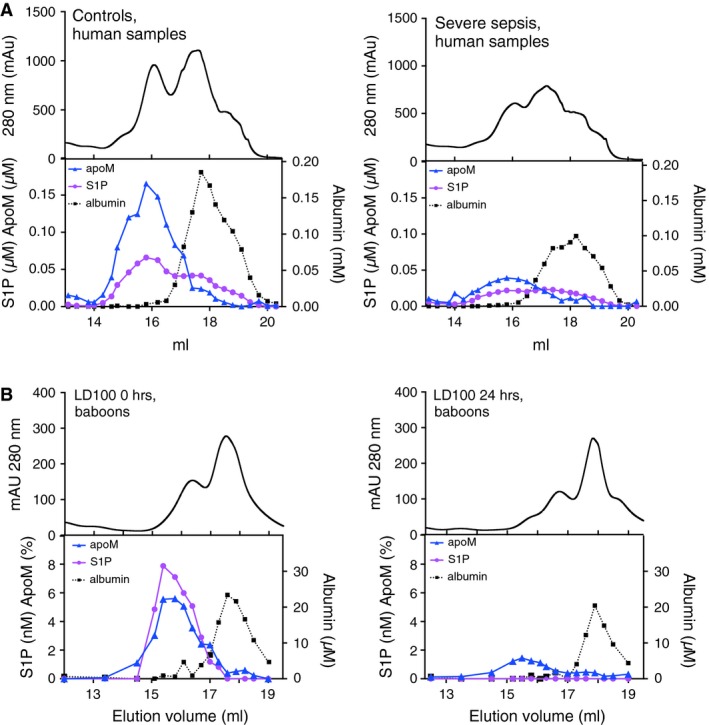
ApoM and S1P co‐elute upon gelfiltration of plasma from healthy and septic humans and baboons. Plasma was pooled from healthy controls (*n* = 10) or patients with severe sepsis (*n* = 10) and subjected to gel filtration analysis on a Superose 6 column (A). Plasma samples drawn from baboons in the LD_100_ group at time‐point zero and 24 hrs post‐infusion were pooled separately and subjected to gel filtration chromatography as above (B). The top panel shows the total protein content (measured by absorbance at 280 nm) and the lower panel shows the content of S1P, apoM and albumin in the eluted fractions.

To investigate whether S1P during sepsis was lost from both apoM and albumin, plasma samples from human controls, human patients with severe sepsis, LD_50_ and LD_100_ septic baboons were subjected to gel filtration chromatography. Confirming our earlier published results 11, around 60% of S1P was bound to apoM and 40% to albumin in human controls (Fig. 7A). However, in patients with severe sepsis, S1P was distributed equally between apoM and albumin (Fig. 7A), indicating that during sepsis S1P is mainly lost from apoM.

In normal baboon plasma, essentially all S1P eluted in the apoM‐containing fractions well separated from the albumin peak (Fig. 7B). Upon sepsis induction in both the LD_50_ (Fig. S3) and LD_100_ groups, the apoM‐associated S1P peak decreased, being too low to be measurable in the LD_100_‐group (Fig. 7B). No translocation of S1P to the albumin‐fractions could be observed.

## Discussion

Sepsis is the leading cause of death in intensive care units and affects approximately 31 million individuals per year worldwide 1. Treatment options are limited and basic mechanisms involved need to be further clarified. S1P has recently emerged as an interesting diagnostic and therapeutic target, not only in sepsis but also in viral infections 28, 33. This study aims at elucidating the response of the endothelial‐protective S1P and its carrier protein apoM to the inflammatory challenge associated with sepsis and SIRS. We investigated in detail the time‐dependent effects on these molecules as well as factors regulating their expression and secretion in disease. In a human sepsis cohort, previously studied for apoM, we found disease‐severity correlated decrease in plasma S1P levels, the profile mimicking that of plasma apoM. To further characterize changes in S1P and apoM during sepsis we have investigated archived plasma and tissue samples from a well‐characterized *E. coli* sepsis model in baboons 37. A major advantage with this model is that immunological reagents developed for humans recognize the baboon counterparts as a result of the high degree of genetic similarity. Similar to the human sepsis cohort, where the S1P and apoM levels decreased in relation to the severity of disease, baboons with the most severe disease had the most pronounced decrease in S1P and apoM. Importantly, the decrease in S1P and apoM observed in the baboon sepsis model, which is based on induction of acute bacteraemia, mimicked that observed in the human cohort, where a natural disease progression occurred.

This shows that the levels of in particular S1P reflect the severity of the disease and being a very important molecule for the endothelial integrity, its decrease possibly contributes to the increased vascular leakage.

In human plasma, the concentrations of S1P and apoM are both around 0.9 μM and approximately 60% of S1P is carried by apoM and 40% by albumin. As judged by the gel filtration analysis, baboons are different in having all their S1P bound to HDL‐associated apoM and no albumin/S1P complexes. In the baboon sepsis model, the decrease in plasma S1P was observed at earlier time‐points than the decrease in apoM, indicating that the S1P‐apoM interaction is dynamic and the molecules have distinct clearance pathways. These results also suggest that it is not the decrease in apoM *per se* that induces a decrease in plasma S1P as has been suggested by others 28, 33. The reduced S1P levels we observed could be because of increased degradation/consumption or decreased synthesis/secretion from S1P‐producing cells, for example platelets, erythrocytes and endothelial cells. Erythrocytes are believed to be the most important source of S1P in plasma and the excessive eryptosis (apoptosis of erythrocytes) that occurs during fever and sepsis 46 may be a reason for decreased S1P‐levels. However, in the baboon model erythrocytes decreased later than S1P and we observed no correlation between erythrocyte numbers and S1P. Instead platelet levels followed closely the pattern of S1P and S1P correlated very well with platelets in both human and baboon sepsis, which agrees well with recent observations made in dengue fever 28. Thrombocytopenia is common in sepsis and is an independent predictor of mortality 45. Whether there is a causal relationship between the thrombocytopenia and the decreased S1P levels in sepsis remains to be elucidated.

In the septic baboons, the liver mRNA levels of apoM, apoA1, apoE and of albumin decreased to very low levels demonstrating that they behave as negative acute phase proteins with decreased synthesis during inflammatory challenges. Interestingly, apoM and apoE mRNA decreased also in the kidney during the septic challenge. The apoM‐gene is located in the major histocompatibility complex (MHC) Class III region on human chromosome 6p21.3. Together with apoM, seven genes (*e.g*. the TNF Family and BAT3) within MHC III are believed to be involved in the inflammatory response 48. The potential role of apoM in immune defence is not obvious but it has been indicated to be protective against atherosclerosis and to inhibit vascular endothelial inflammation 49, 50. Regulation of the apoM‐gene during sepsis has not been analysed in this study but it might be achieved by alterations in, for example, TGF‐β and HNF1, proteins suggested to regulate apoM expression and which have been demonstrated to be increased and decreased, respectively, in sepsis 51, 52, 53, 54, 55. ApoA1, apoE and albumin also demonstrated significantly decreased plasma levels in the severely sick animals. However, in the surviving animals, plasma levels of apoE and to a lesser extent also apoM tended to increase at the later time‐points. We have no experimental explanation for this late increase, as liver tissues were not available after 34 hrs. In humans, plasma levels of apoE are reportedly increased during severe inflammation 39, 56. This increase in apoE may be because of a decreased expression of apoE‐receptors (low‐density lipoprotein receptor (LDLr) and low‐density lipoprotein receptor‐related protein) with lowering of the clearance of apoE 57. LDLr^−/−^ mice have increased plasma levels of apoM and apoE 54. Moreover, in LDLr‐overexpressing mice, apoM and S1P levels are both decreased, an effect which is abolished when additionally knocking out apoE in the LDLr‐overexpressing mice 58. This indicates that apoE and apoM may be eliminated *via* similar pathways.

In conclusion, our results demonstrate that S1P and apoM are strongly reduced in both human and non‐human primate sepsis, the degree of decrease in concentration reflecting the severity of the disease. The decrease in S1P may contribute to the pathogenic mechanisms of the disease, in particular to the increased vascular leakage. The strong correlation between the decreased S1P‐levels and disease severity may potentially have clinical value in terms of prognostic evaluation.

## Conflict‐of‐interest

The authors have no competing financial interests or other conflicts of interest to declare.

## Supporting information


**Fig. S1** Transcription of enzymes involved in S1P‐metabolism is altered in the septic liver.

**Fig. S2** Plasma concentrations of *E. coli* correlate with the decrease in S1P.

**Fig. S3** ApoM and S1P co‐elute upon gel filtration of plasma from healthy and septic LD50‐baboons.
