# Supplementary figures and images for: Sphingosine 1‐phosphate and its carrier apolipoprotein M in human sepsis and in Escherichia coli sepsis in baboons
Source: J Cell Mol Med. 2016 Mar 17;20(6):1170–81. doi: 10.1111/jcmm.12831 (PMC4882985; doi:10.1111/jcmm.12831)

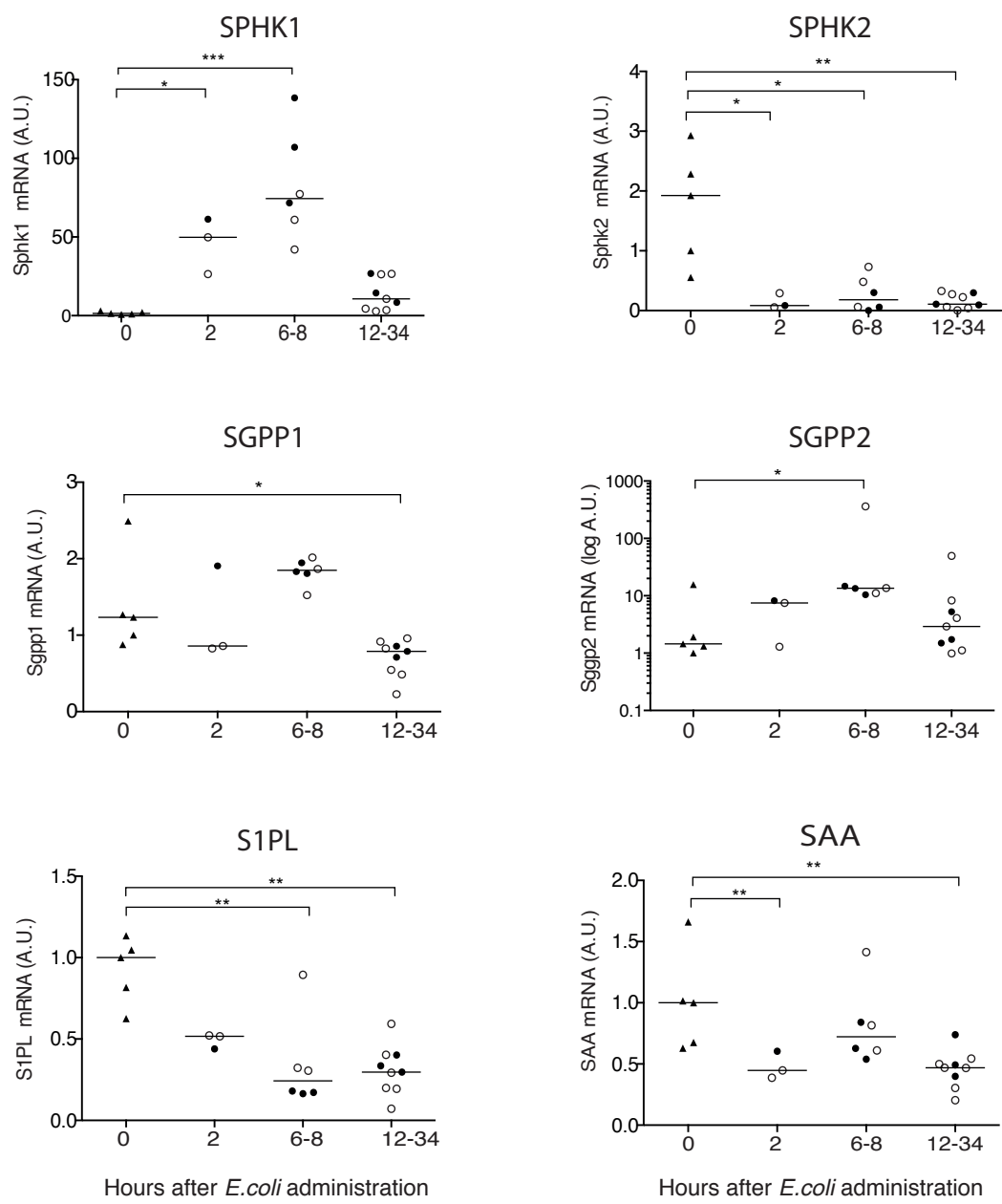

Supplement: Supplementary file 1 — Fig. S1 Transcription of enzymes involved in S1P‐metabolism is altered in the septic liver. [file JCMM-20-1170-s001.pdf]

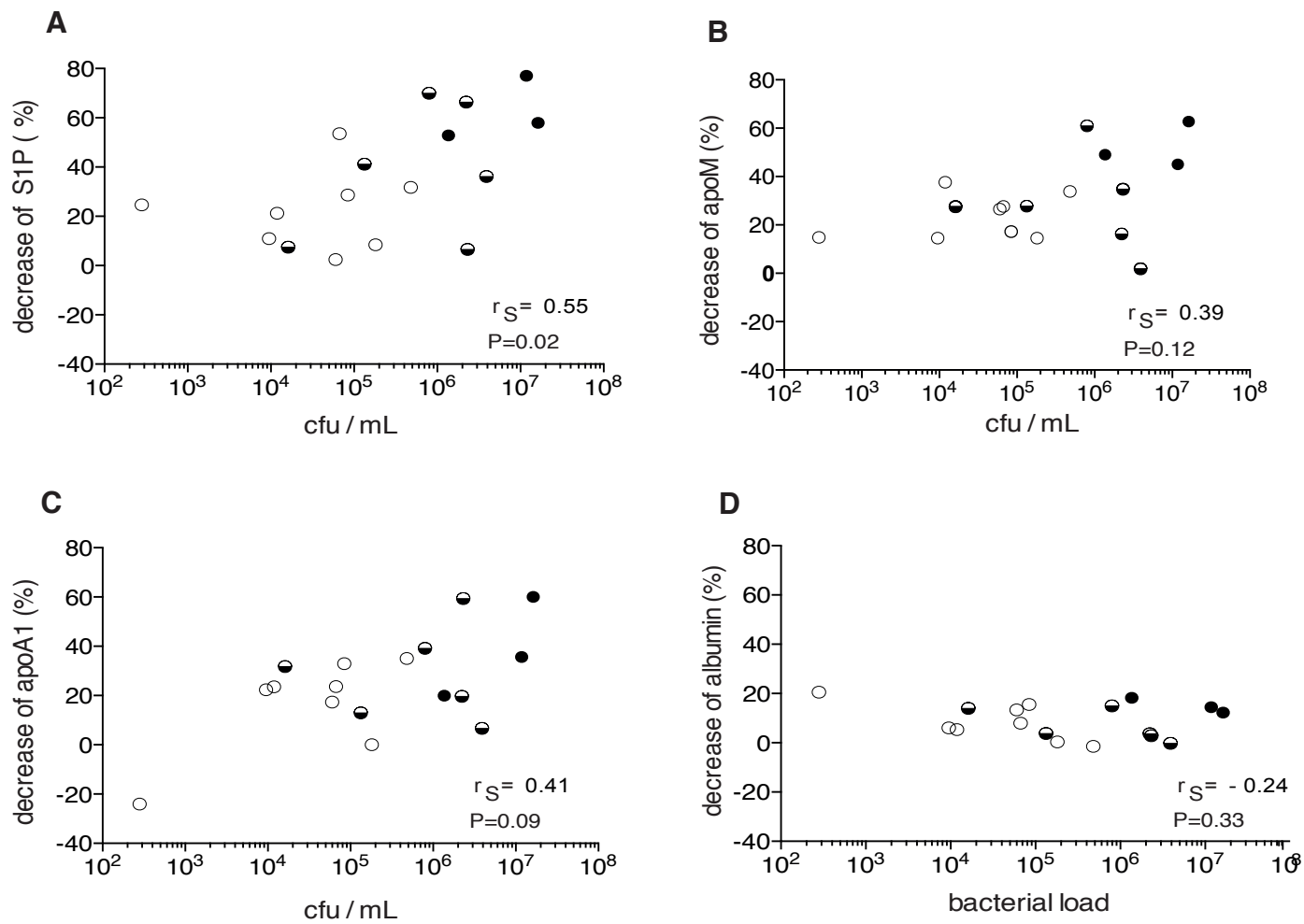

Supplement: Supplementary file 2 — Fig. S2 Plasma concentrations of E. coli correlate with the decrease in S1P. [file JCMM-20-1170-s002.pdf]

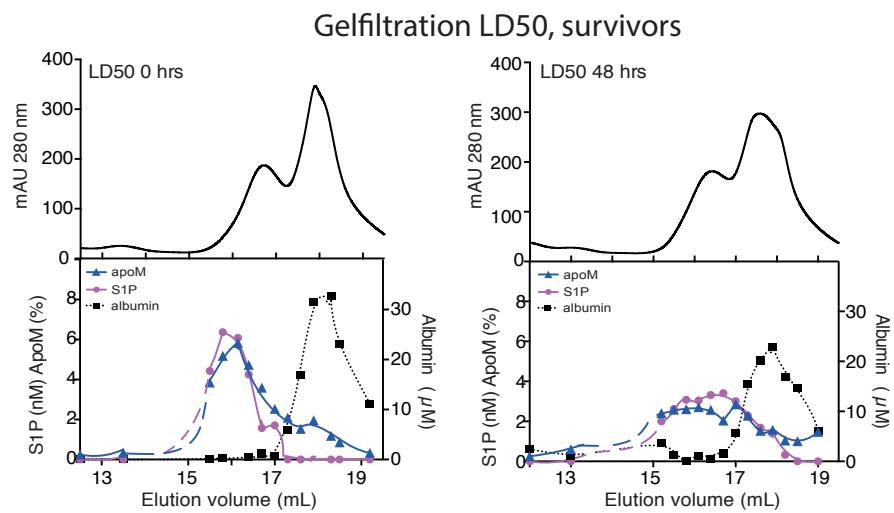

Supplement: Supplementary file 3 — Fig. S3 ApoM and S1P co‐elute upon gel filtration of plasma from healthy and septic LD50‐baboons. [file JCMM-20-1170-s003.pdf]
